# Medical Opinion and Movement

**Published:** 1906-08-11

**Authors:** 


### Medical Opinion and Movement.

The Home Secretary has appointed a departmental committee to inquire and report what diseases and injuries, other than injuries by accident, are due to industrial occupations, are distinguishable as such, and can properly be added to the diseases enumerated in the third schedule of the Workmen's Compensation Bill, 1906, so as to entitle to compensation persons who may be afflicted thereby. Mr. Herbert Samuel, M.P., is the Chairman, and the other members are Professor Clifford Allbutt, Mr. H. H. Cunynghame, C.B., and Dr. T. M. Legge. The secretary is Mr. F. L. B. Elliott of the Home Office, to whom correspondence should be addressed.

The meetings of the various learned and scientific societies which are now being held exhibit an interest which they sometimes lack. It is worthy of note, for example, that at the celebration at the Royal Institution of the fiftieth anniversary of the discovery of aniline dyes it was pointed out that this discovery, so far as its commercial value was concerned, was overlooked by the English community. Foreign chemists were much more commercially alert than those in England, with the result that a great industry has been practically lost to the British people, for aniline dyes are mainly manufactured at the present time by foreigners. Another curious sidelight on this question is supplied by the fact that Sir William Perkins made his discovery of the mauve colour to be derived from tar when he was searching for the means to produce quinine artificially.

No full report of the proceedings of the British Association has been forthcoming until the present week, owing to the great pressure upon the London daily papers and the fact that the rising of Parliament has been unusually expedited this year. In the Chemical Section Professor Wyndham Dunstan called attention to the fact that the physical properties of raw rubber, on which its technical value depends, are to be correlated with the chemical composition of the material. This fact is important from the circumstance that the finest caoutchouc is only yielded by some half-dozen plants, and that the elastic substance in each case presents a very similar, if not identical, chemical structure. The production of caoutchouc by chemical means has been virtually accomplished in its formation from isoprene, although the exact nature of this change has still to be determined. If the cheapening of the cost of production can be secured, the manufacture of synthetic rubber may soon become a purely practical problem. There is at the present time a great extension of rubber planting owing to the growing demand for the material. It seems, therefore, to be probable that a race may soon commence between the manufacturing chemists and the rubber planter for the supply of this material to the markets in the near future.

The President of the Anthropological Section, Mr. E. Sydney Hartland, gave an interesting address on the origin and early relation of magic and religion. The religious practices of savage and barbarous peoples were based upon ideas which we had grouped together under the name of animism. Animism, as Professor Tylor said, is the groundwork of the philosophy of religion, from that of savages up to that of civilised man. Animism, or the distinction of soul and body, was a development necessitated by subsequent observation as civilisation advanced, and the trend of reasoning which that observation awakened. Originally man's attitude was first of all, and intensely, practical, not contemplative. Archaic beliefs died hard, and in remote parts of England it is still firmly believed that a witch might assume the form of a hare which, if wounded by a sportsman, would afterwards be found on the witch's proper person, testifying beyond dispute to the preservation of her individuality under the change of shape and species. We thus arrive at shape shifting, as it was called, which might even take place by means of death and a new birth without the loss of identity.

In West Africa the belief in a new birth without loss of identity is proved by the fact that when a baby arrives in a family it is shown a selection of small articles belonging to deceased members, and the thing which the child catches hold of identifies his as "Uncle John" or "Cousin Emma," and so forth. So far as this belief prevails it is held by some that garments once worn, or other objects which have been in intimate contact with a human being, are penetrated by his personality, and remain, as it were, united with him for good or ill. Mr. Hartland contends that such beliefs as these exhibit a concept of personality imperfectly crystallised. In nearly all stages of civilisation now to be found in the world, what we call supernatural beings were concerned with the initiation of the magician. The schism between magic and religion was a latter development of civilisation. When it occurred, as the history of heresy in Europe and the witch trials teach, it was rather magic in its anti-social aspect than in itself which was reprobated and punished. It is strange in this connection to notice that the magician was only condemned when he departed from established custom and established beliefs which involved a severance from the community and an imputation of anti-social ends. Practices essentially magical might be incorporated in religious rites and exercised for what was believed to be the general good. In such a case they have continued to be exercised with general assent in the highest forms of religion.

